# Extensor tenosynovitis and subcutaneous abscess caused by hypervirulent *Klebsiella pneumoniae*

**DOI:** 10.1016/j.idcr.2025.e02427

**Published:** 2025-11-10

**Authors:** Takahiro Fukushima, Takaaki Kobayashi, Poorani Sekar, Shinichi Sotome, Tadashi Eguchi, Akihito Yoshida

**Affiliations:** a Department of General Internal Medicine, Kameda Medical Center, Kamogawa, Chiba, Japan; b Division of Infectious Diseases, University of Kentucky College of Medicine, Lexington, KY, USA; c Division of Infectious Diseases, University of Iowa College of Medicine, Iowa City, IA, USA; d Department of Orthopedic Surgery, Kameda Medical Center, Kamogawa, Chiba, Japan

**Keywords:** Hypervirulent *Klebsiella pneumoniae*, Tenosynovitis, Subcutaneous abscess, Liver abscess

## Abstract

We report a rare case of invasive *Klebsiella pneumoniae* infection in a previously healthy 52-year-old Japanese man who presented with extensor tenosynovitis associated with subcutaneous abscess of the right hand, in the setting of concomitant liver and prostatic abscesses. Cultures from blood, liver abscess aspirate, synovial fluid and aspirate from the subcutaneous tissue of the hand all grew *K. pneumoniae*. Although genomic biomarker testing was not available, a positive string test along with the characteristic clinical presentation suggested a hypervirulent phenotype. Surgical debridement of the hand and targeted antimicrobial therapy led to clinical improvement. This case underscores the importance of considering hematogenous dissemination in cases of tenosynovitis without a history of trauma and highlights the protean manifestations of hypervirulent *K. pneumoniae*.

## Introduction

Hypervirulent *Klebsiella pneumoniae* (hvKp) is a pathotype distinct from classic *K. pneumoniae* (cKp), known for its ability to cause invasive, community-onset infections in otherwise healthy individuals. First described in East Asia, particularly Taiwan, hvKp has become a significant global pathogen over the past two decades [1]. While cKp primarily causes nosocomial infections such as pneumonia, urinary tract infections, and bacteremia in immunocompromised patients, hvKp has a unique propensity to cause metastatic infections, including liver abscess, endophthalmitis, meningitis, and osteomyelitis—even in the absence of traditional risk factors [2].

HvKp strains possess virulence-enhancing genetic elements, often located on large plasmids. These include regulators of capsule production (*rmpA*, *rmpA2*), siderophore systems (*iucA*, *iroB*), and other factors like *peg-344*, which enhance mucoviscosity, immune evasion, and nutrient acquisition [3]. Clinically, these strains are often associated with a hypermucoviscous phenotype detectable via the "string test," in which colonies produce strings ≥ 5 mm when stretched by an inoculation loop [4]. However, this phenotypic test is neither specific nor sensitive and should be interpreted with clinical and microbiological context.

While hvKp-associated liver abscess is well described, extrahepatic manifestations involving the soft tissue and musculoskeletal system are relatively uncommon. Necrotizing fasciitis is the most well-recognized soft tissue presentation of hvKp; however, other manifestations, such as tenosynovitis, have not previously been reported. Pyogenic tenosynovitis typically affects the flexor tendons, and is usually caused by direct inoculation via trauma, bites, or puncture wounds with hematogenous spread being rare [5] [6]. In this report, we describe a rare case of hvKp presenting as extensor tenosynovitis of the hand with associated subcutaneous abscess, in the context of disseminated infection involving concurrent hepatic and prostatic abscesses.

## Case presentation

A 52-year-old Japanese man with a medical history of vasospastic angina, dyslipidemia, hypertension, and ventricular fibrillation presented to a clinic with one week of generalized fatigue, followed by increasing right-hand pain and swelling over the past two days. He denied any trauma, recent injections, or known insect bites to the hand. He was initially managed symptomatically with acetaminophen, but his symptoms progressively worsened, prompting further evaluation.

**Fig. 1:**
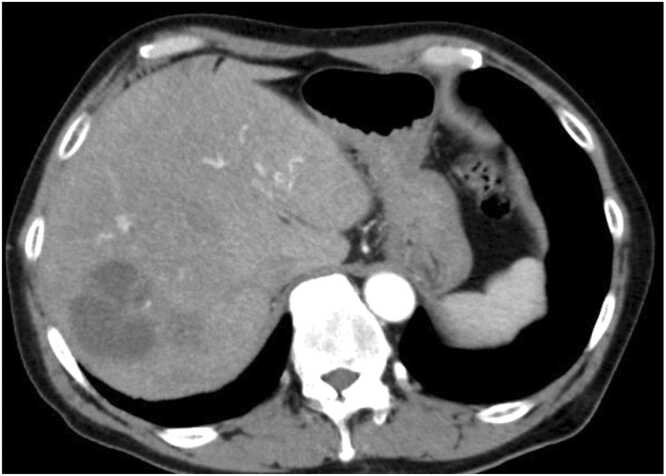
A contrast-enhanced CT scan of the abdomen. A multiloculated, 5 cm hypoattenuating lesion in the right hepatic lobe is noted, consistent with a liver abscess.

On presentation to an outside facility, he was febrile and tachycardic. Laboratory testing revealed leukocytosis with neutrophil predominance. Contrast-enhanced abdominal computed tomography (CT) scan showed a multiloculated, 5 cm hypoattenuating lesion in the right hepatic lobe, consistent with a liver abscess (Fig. 1). Empirical intravenous ampicillin-sulbactam was initiated, and he was transferred to a tertiary care center.

At the tertiary care center, he had a temperature of 37.6°C, heart rate of 94 beats/minute, respiratory rate of 18 breaths/minute, and blood pressure of 103/72 mm Hg. His right hand was swollen from the wrist through the dorsum of the hand. Physical examination revealed tenderness over the dorsum of the right hand and limited range of motion of the wrist. Pain was also elicited with passive extension of the digits. The remainder of his physical exam was unremarkable. Laboratory workup indicated a white blood cell count of 15.8 K/μL with neutrophilia and a C-reactive protein (CRP) level of 15.98 mg/dL (reference range 0–0.14 mg/dL). His liver function test was elevated with an alanine aminotransferase (ALT) of 63 U/l (reference range 0–33 U/l), aspartate aminotransferase (AST) of 59 U/l (reference range 0–32 U/l), gamma-glutamyl transferase (GGT) of 260 U/l (reference range 0–40 U/l), and bilirubin of 1.2 mg/dL (reference range 0.2–1.3 mg/dL). Ultrasound of the hand showed a fluid collection in the subcutaneous tissue without joint effusion. Aspiration of the collection yielded purulent fluid, which demonstrated Gram-negative rods on microscopy. Blood cultures obtained on admission subsequently grew Gram-negative rods. Given evidence of multi-organ involvement, hypervirulent *K. pneumoniae* infection was suspected. Contrast-enhanced CT of the brain and ophthalmologic evaluation ruled out brain abscess and endophthalmitis. A percutaneous drainage catheter was placed in the liver abscess, and antibiotics were changed to ceftriaxone and metronidazole.

**Fig. 2:**
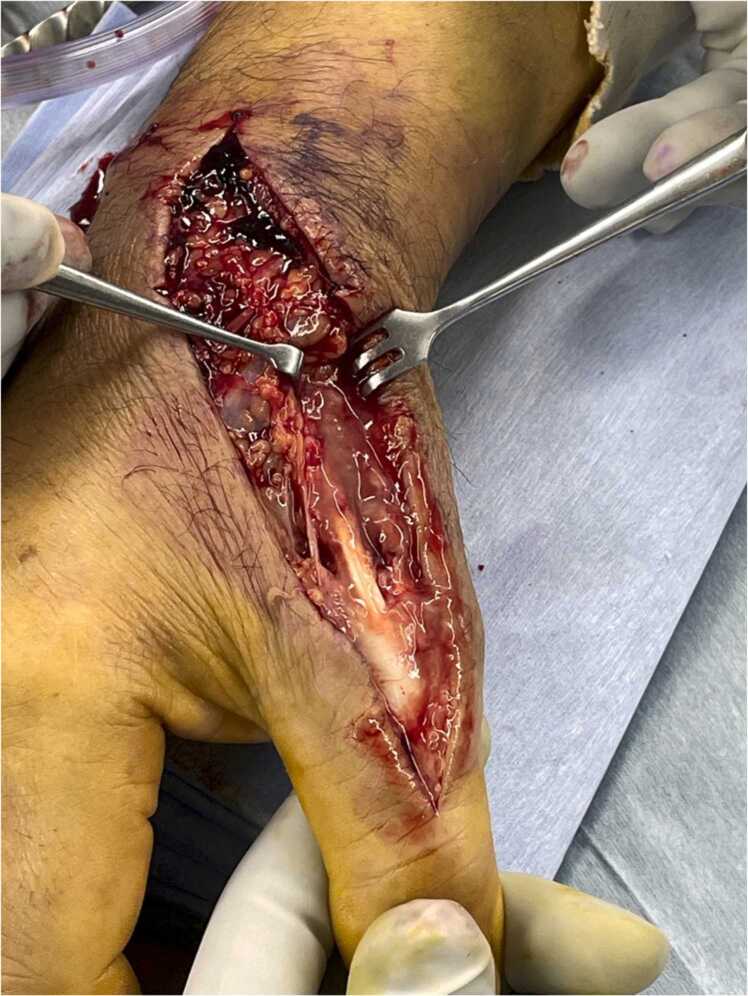
Surgical debridement of the right hand. Purulent exudate was noted along the extensor pollicis longus tendon distal to the metacarpophalangeal joint.

**Fig. 3:**
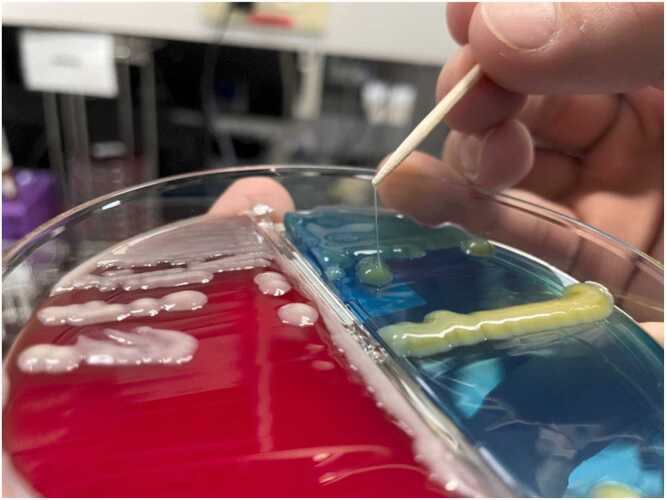
Colony of *K.pneumoniae*. The isolate produced a positive string test.

Despite antimicrobial therapy, swelling of the right hand persisted. Magnetic resonance imaging (MRI) revealed tenosynovitis of the extensor pollicis longus tendon with residual subcutaneous abscess. Surgical exploration was performed on hospital day 1. Purulent fluid was present, tracking along the tendon sheath of the extensor pollicis longus distal to the metacarpophalangeal joint (Fig. 2). No other tendon compartments or joints were involved. Debridement of the infected tendon sheath and subcutaneous abscess was subsequently performed. Cultures from the liver abscess aspirate, subcutaneous tissue and synovial fluid of the hand, and blood all grew *K. pneumoniae*. The isolate produced a positive string test (>5 mm), suggestive of the hypermucoviscous phenotype (Fig. 3). Susceptibility testing revealed that the isolate was susceptible to third-generation cephalosporins, including ceftriaxone. Metronidazole was discontinued. Molecular characterization was not available, but the clinical syndrome and phenotypic features supported the diagnosis of hvKp.

On day 10, repeat abdominal imaging revealed a hypodense lesion in the prostate. Aspiration of the lesion confirmed a prostatic abscess, also culture-positive for *K. pneumoniae*. Due to development of neutropenia, ceftriaxone was replaced with cefotaxime. The patient’s clinical status gradually improved, and he was discharged on hospital day 36 on oral ciprofloxacin. Ciprofloxacin was continued until resolution of the liver abscess. At subsequent follow patient continued to do well with relatively preserved range of motion of the hand and no recurrence of infection.

## Discussion

This case demonstrates a rare presentation of hvKp causing extensor tenosynovitis with a subcutaneous abscess of the hand in an immunocompetent host. Tenosynovitis is most often caused by direct inoculation following trauma or animal bites and typically involves the flexor tendons of the hand [7]. It carries significant morbidity including tendon rupture and functional impairment, necessitating prompt recognition and surgical intervention. Hematogenous seeding of tendon sheaths is rare, though previously described in cases involving *Neisseria gonorrhoeae*, *Staphylococcus aureus*, and *Streptococcus pyogenes* [5], [6]. In this case, the absence of a cutaneous portal of entry and concurrent hepatic and prostatic abscesses strongly suggests hematogenous spread.

Extensor tenosynovitis is a rare clinical entity, with the existing literature limited to case reports and a few small case series. Previous studies have frequently identified atypical microorganisms, such as Mycobacterium species (including both tuberculous and nontuberculous mycobacteria) and fungi, whereas reports of typical bacterial pathogens are relatively uncommon [8], [9]. *Neisseria gonorrhoeae* is the most frequently reported pathogen, typically in the context of disseminated gonococcal infection, suggesting hematogenous spread [10]. In a single-center study, all 20 reported cases of extensor tenosynovitis were associated with intravenous drug use, with *Staphylococcus aureus* being the most commonly isolated pathogen [11]. While some cases have been attributed to trauma, such as animal bites, idiopathic cases without an identifiable inciting event have also been described [12].

HvKp has a known predilection for causing liver abscesses and distant metastases. The combination of bacteremia, liver abscess, and soft tissue involvement, in the absence of trauma, is a classic but underrecognized presentation of hvKp. The isolate in this case demonstrated a positive string test, a commonly used phenotypic marker for hypermucoviscosity. However, the string test is not a definitive diagnostic tool. One study reported a sensitivity of 0.91 and a specificity of 0.89, suggesting that this test is suboptimal for distinguishing hvKp from classical *K. pneumoniae* strains [13]. Recently, the presence of all five genotypic biomarkers *— peg-344*, *rmpA*, *rmpA2*, *iroB*, and *iucA* —has been reported to be much more accurate in distinguishing hvKp from cKp [14]. Although these diagnostic biomarkers were unavailable in our hospital, positive string tests and clinical features of community-acquired infection with multifocal abscesses involving the liver strongly suggest hvKp as the causative organism.

Hypervirulence of hvKp is attributable to various genetic factors, notably those enhancing capsular polysaccharide production and iron acquisition. These features contribute to resistance to phagocytosis and complement-mediated killing, promoting dissemination even in immunocompetent individuals [15].

While hvKp strains are typically susceptible to cephalosporins, there are increasing reports of strains that harbor resistance genes, including extended-spectrum beta-lactamases (ESBLs) and carbapenemases [16], [17]. The emergence of such strains present a dual threat: hypervirulence and multidrug resistance [18]. In this case, the isolate remained susceptible, and the patient responded well to ceftriaxone followed by oral ciprofloxacin.

Treatment of hvKp infections hinges on timely diagnosis, effective source control, and appropriate antimicrobial therapy. In our patient, multiple drainage procedures (surgical and percutaneous) were necessary. Recurrence is a well-recognized phenomenon in hvKp infection, particularly if treatment is prematurely stopped or metastatic foci are missed [19]. Prolonged therapy and close follow-up with imaging is often warranted.

To our knowledge, this is the first published case of hvKp-associated tenosynovitis. Our case highlights a novel site of infection (extensor tenosynovitis) with hvKp. It also reinforces the need for clinicians to consider this diagnosis even in soft tissue infections when the history lacks a clear inciting event. Such recognition is crucial for ensuring proper management and preventing missed sites of dissemination.

## Author statement

All authors have made substantial contributions to one or more of the following aspects of the work:

・The conception and design of the study, acquisition of data, or analysis and interpretation of data;

・Drafting the manuscript or revising it critically for important intellectual content;

・Final approval of the version to be submitted.

We confirm that Takahiro Fukushima has been designated as the corresponding author and will be responsible for all communication with the editorial office during the submission and review process. All authors agree to be accountable for all aspects of the work to ensure that any questions related to the accuracy or integrity of any part of the work are appropriately investigated and resolved.

## Author contributions

Takahiro Fukushima wrote the first draft of the manuscript. Takaaki Kobayashi, Poorani Sekar, Shinichi Sotome, Tadashi Eguchi and Akihito Yoshida critically reviewed and revised the manuscript. All authors read and approved the final paper

## CRediT authorship contribution statement

**Shinichi Sotome:** Writing – review & editing. **Poorani Sekar:** Writing – review & editing. **Akihito Yoshida:** Writing – review & editing. **Tadashi Eguchi:** Writing – review & editing. **Takahiro Fukushima:** Writing – original draft, Conceptualization. **Takaaki Kobayashi:** Writing – review & editing.

## Ethical approval

The patient provided informed consent for the publication of the clinical case details.

The study is a case report, only information from the patient’s file was used, no type of intervention was performed with the patient, so it does not have approval from the ethics committee

## Funding

This research did not receive any specific grant from funding agencies in the public, commercial, or not-for-profit sectors.

## Declaration of Competing Interest

The authors declare that they have no known competing financial interests or personal relationships that could have appeared to influence the work reported in this paper.
